# Sarcopenia in older adults admitted to acute medical unit: a prospective observational study of feasibility and clinical outcomes

**DOI:** 10.3389/fmed.2026.1862604

**Published:** 2026-08-20

**Authors:** Vicky Kamwa, Zaki Hassan-Smith, Thomas Jackson, Elizabeth Sapey

**Affiliations:** 1 Birmingham Acute Care Research Group, Department of Inflammation and Ageing, University of Birmingham, Birmingham, United Kingdom; 2 Aston Medical School, Aston University, Birmingham, United Kingdom; 3 Department of Inflammation and Ageing, University of Birmingham, Birmingham, United Kingdom; 4 The Health Data Research UK Hub in Acute Care (PIONEER), Birmingham, United Kingdom

**Keywords:** acute medical unit, aging, older adults, sarcopenia, trajectory, feasibility study

## Abstract

**Background:**

Sarcopenia, the loss of skeletal muscle mass and function, has recently emerged as a significant health concern. It is common in older adults and linked to poor outcomes. It is dynamic and can improve or deteriorate over short periods of time. We aimed to determine its prevalence and changes during hospital admission and explore associations with clinical outcomes.

**Methods:**

This was a Prospective, Observational Cohort, Feasibility Study. Older Adults (>65 years old) with an unplanned admission to an AMU in a secondary care hospital were eligible to participate in the study. Muscle mass was measured using an ultrasound, handgrip strength using a hand-held dynamometer and 4 meters gait speed on admission, at day 3 and day 7. Outcomes were assessed up to 3 months, and we recorded mortality at 6 months.

**Results:**

A total of 106 patients were recruited from 232 screened. Only 18 participants completed 3-month follow up although 6-month mortality was recorded for all participants. Mean (SD) age at admission was 76.8 years (7.0); 56% were female and 97.2% White. Sarcopenia prevalence was 60.4% on admission. Patients with Sarcopenia were nearly 6 times (OR 5.94, 95% CI 1.66–28.6) more likely to die within 6 months, including when adjusted for nutritional status and hemoglobin. Low muscle mass, strength, and gait speed, as individual factors on admission, were also associated with 6-month mortality (Correlation co-efficient *R* = −0.67, *p* < 0.001; *R* = −0.76, *p* < 0.001 and *R* = −0.54, *p* < 0.001, respectively). Longitudinal data were limited due to study withdrawal and exploratory sarcopenia trajectories varied across individuals who remained in the study.

**Conclusion:**

Sarcopenia is highly prevalent among older adults acutely admitted to hospital; and although worse health outcomes, including mortality, were observed in those with sarcopenia, these findings are exploratory. Delivering longitudinal research in this population remains challenging, particularly due to high withdrawal rates in less robust adults.

## Introduction

Sarcopenia is a condition associated with poor muscle function, defined as a state of low muscle strength (indicative of probable sarcopenia) and muscle mass (confirmed sarcopenia) (1). Sarcopenia is common, especially in older adults, with an estimated prevalence varying between 6.8% and 7.8% in community-dwelling older adults (2), which increases to 17.7%–73.3% in care home residents and 22%–87% in assisted-living facilities (3). A recent systematic review and meta-analysis also found a prevalence of between 8% and 36% in individuals <60 years and ranging from 10% to 27% in ≥60 years (4).

The causes for sarcopenia are diverse and include immobility or muscle disuse, medical conditions linked to catabolic and anabolic imbalance (5), and persistent inflammation such as raised Tumor Necrosis Factor α (TNF-α), C-reactive protein (CRP), and Interleukin (IL)-6 or low alpha1-antichymotypsin (ACT) (6–8). Sarcopenia is associated with a greater risk of polypharmacy (9) and polypharmacy itself and potentially inappropriate medications use are strongly associated with new-onset sarcopenia in community-dwelling older adults (10).

Sarcopenia is an important condition to recognize as it is associated with numerous adverse outcomes such as falls, cognitive impairment (11), delirium (12) and mortality (13, 14). The incidence of sarcopenia also correlates with admissions to hospital and the number of days spent in bed during that admission (15). Importantly, sarcopenia may be treatable with studies suggesting improvements in physical performance and increases in muscle strength and power with exercise programs or physical therapy (16, 17), nutritional support (18, 19), and interventions to address underlying causes. The gold standard methods for diagnosing sarcopenia remain either DXA (bone densitometry) scanning, CT or MRI scans of the skeletal muscle (1, 20). However, scanning capacity is limited. More recently ultrasound has been used as a research tool, with studies showing high reproducibility when carried out under appropriate conditions by trained individuals (21, 22).

A recent systematic review reported evidence supporting the importance of diagnosing sarcopenia and frailty in acute medical units to enable early identification and targeted interventions (23). However, studies were heterogeneous in design, with small sample sizes. The review suggested there was a need for more studies to better understand the prevalence of sarcopenia in acute medical units, and to document those most at risk of poor outcomes when frailty was diagnosed. There has been increasing evidence of an association between low Mini-Nutritional Assessment (MNA) score (<24), diagnosis of malnutrition and the development of sarcopenia (24, 25). Growing evidence has also recently appeared around the association between sarcopenia and low level of hemoglobin across the globe and in patients with diverse organ-diseases (26–29).

To date and to be best of our knowledge, no studies have assessed dynamic changes in sarcopenia over the course of an admission, as well as longer term outcomes.

We hypothesized that it would be feasible and acceptable to measure sarcopenia in older adults admitted to hospital unexpectedly. Further, that the condition would be common, would worsen over the course of the admission and would be associated with poorer clinical outcomes. We report here the results of the feasibility study.

## Materials and methods

### Study design and study population

The study protocol has been published (30) and been ethically approved by the relevant bodies including the Health Research Authority (IRAS 279017; REC 20/WA/0263 and RG_20-076). This was a single-center prospective, observational study including patients aged ≥65 years admitted to an acute medical unit (AMU) within 72 h. It was conducted in a large, urban, NHS hospital: the Queen Elizabeth Hospital Birmingham (QEHB) in the United Kingdom (UK), from October 2021 to December 2022.

Patients aged less than 65 years, patients declining consent or for patients without capacity where the personal or professional consultee declined participation and patients with palliative diagnosis not expected to survive to discharge were all excluded from the study.

### Objectives

The study objectives were threefold. The first objective was to determine the feasibility and acceptability of repeated sarcopenia assessments in acutely admitted medical patients, on admission, at day three and day seven and at 3 month follow up; The second was to assess the change in sarcopenia over the acute admission. The third objective was to determine whether the presence of sarcopenia at admission, or the change in this condition can predict adverse outcomes.

### Data collection

Demographics, medical data and an assessment of sarcopenia was recorded on day 0 of the study, day 3, day 7 and at month 3. Outcomes were assessed during the admission and 3 months following discharge. We also assessed mortality at 6 months from hospital and primary care records.

### Outcomes

#### Primary

The primary outcome for the feasibility study was the number of participants who agreed to data collection and an assessment of sarcopenia at each study visit.

#### Secondary

Secondary outcomes included length of stay, 7-day and 30-day readmission, in-patient mortality and death at 6 months. These were collected from medical records of participants who had given consent.

### Sarcopenia assessment

In this study, sarcopenia was diagnosed combining assessments of muscle strength and muscle mass, and physical performance was used to determine severity in accordance with the revised EWGSOP2 (1). Confirmed sarcopenia was diagnosed in the presence of low muscle strength with associated low muscle mass. A record of low physical performance as well as sarcopenia conferred the diagnosis of severe sarcopenia. Participants with low muscle strength without low muscle mass were diagnosed as probable sarcopenia and included as a separate group during data analysis. Patients received standard care and no interventions were instituted.

#### Muscle mass measurement

Muscle mass was measured using an ultrasound machine and following the Bilateral Anterior Thigh Thickness (BATT) protocol (21). In brief, the BATT protocol involves measuring the thickness of rectus femoris and vastus intermedius bilaterally; the thickness measurements were summed to calculate the BATT.

Low muscle mass was diagnosed if BATT measurement was less than 38.5 mm in women and less than 54.4 mm in men.

#### Muscle strength measurement

Muscle strength was assessed by measuring handgrip strength (HGS) using a hydraulic hand-held dynamometer (Jamar, Performance Health, USA). Two measurements were recorded at all visits (day of admission and follow-up visits) in kilograms (Kg) for the dominant and non-dominant hand. The highest value was used for analysis. Low muscle strength was diagnosed if the highest value of the HGS recorded was <27 Kg (1).

#### Physical performance measurement

Physical performance was measured using the four meters gait speed and recorded in meters/second (m/s). Low physical performance, a sign of severe sarcopenia, was diagnosed if the gait speed was ≤0.8 m/s (1).

### Nutritional assessment

Nutritional status was assessed using the Mini-nutritional Assessment (MNA)–Full form (31) A score >24 equated to a normal nutritional status, a score between 17 and 23.5 indicated at risk of malnutrition and a score <17 indicated malnutrition.

### Covariates

During our interview with patients on recruitment day (D0), we collected data such as age, sex, ethnicity, smoking status (ex-smoker, current smoker or not), Body Mass Index (BMI), calf circumference (CC) and mid-arm circumference (MAC). We also extracted from their general practitioner and/or hospital medical records: co-morbidities (including stroke, myocardial infarction, diabetes mellitus, congestive cardiac failure, chronic obstructive pulmonary disease, cancers from any origin) and number of falls in the last 12 months preceding their admission.

Physiological measures were recorded including early warning score (NEWS2 score), systolic and diastolic pressure (SBP and DBP), blood glucose, heart rate (HR), respiratory rate (RR), temperature, MNA score, activities of daily living score (ADLS), independent activities of daily living score (iADLS).

Blood tests were also recorded including albumin, alanine phosphatase (ALP), urea, sodium, potassium, calcium, white blood cells (WBC), platelets, international normalized ratio (INR), hemoglobin level (Hb), mean corpuscular volume (MCV), C- Reactive Protein (CRP).

Nutritional status (MNA) and hemoglobin level were included *a priori* as adjustment variables due to strong prior evidence of their association with sarcopenia and mortality.

### Statistical analysis and approach to data inclusion

Although an initial sample size of 165 participants was estimated to inform the design of a future definitive study, this feasibility study was not powered to detect statistically significant differences in clinical outcomes. All inferential analyses are therefore exploratory and hypothesis-generating.

If patients were unable to undergo sarcopenia assessments at the baseline visit, reasons for this were recorded (for feasibility reporting) but they were excluded from our analysis. If patients had completed assessment at the baseline visit and were however, unable to take part in subsequent visits or unable to complete all data assessments at subsequent visits, their data were included in further analysis apart from where the participant requested that all data be removed from the study. No imputation methods were applied for missing data. Analyses were conducted on available cases for each outcome and sample sizes therefore vary across models.

Data analysis was conducted using R Studio version 4.2.3.

Descriptive and quantitative analytic tests were used to review the feasibility and outcome aspects of the study. Odd-Ratios (OR) were calculated to determine the impact of the presence of sarcopenia at the time of admission on in-patient, 3-month and 6-month mortality.

Correlation coefficients R were calculated to estimate the probability of dying at 6-month from logistic models including each of the 3 components of muscle function.

Given the limited number of outcome events, multivariable models were restricted to a small number of pre-specified covariates, and results should be interpreted as exploratory due to potential model instability.

No *p*-value was calculated given the exploratory nature of our study.

## Results

### Study feasibility

**FIGURE 1:**
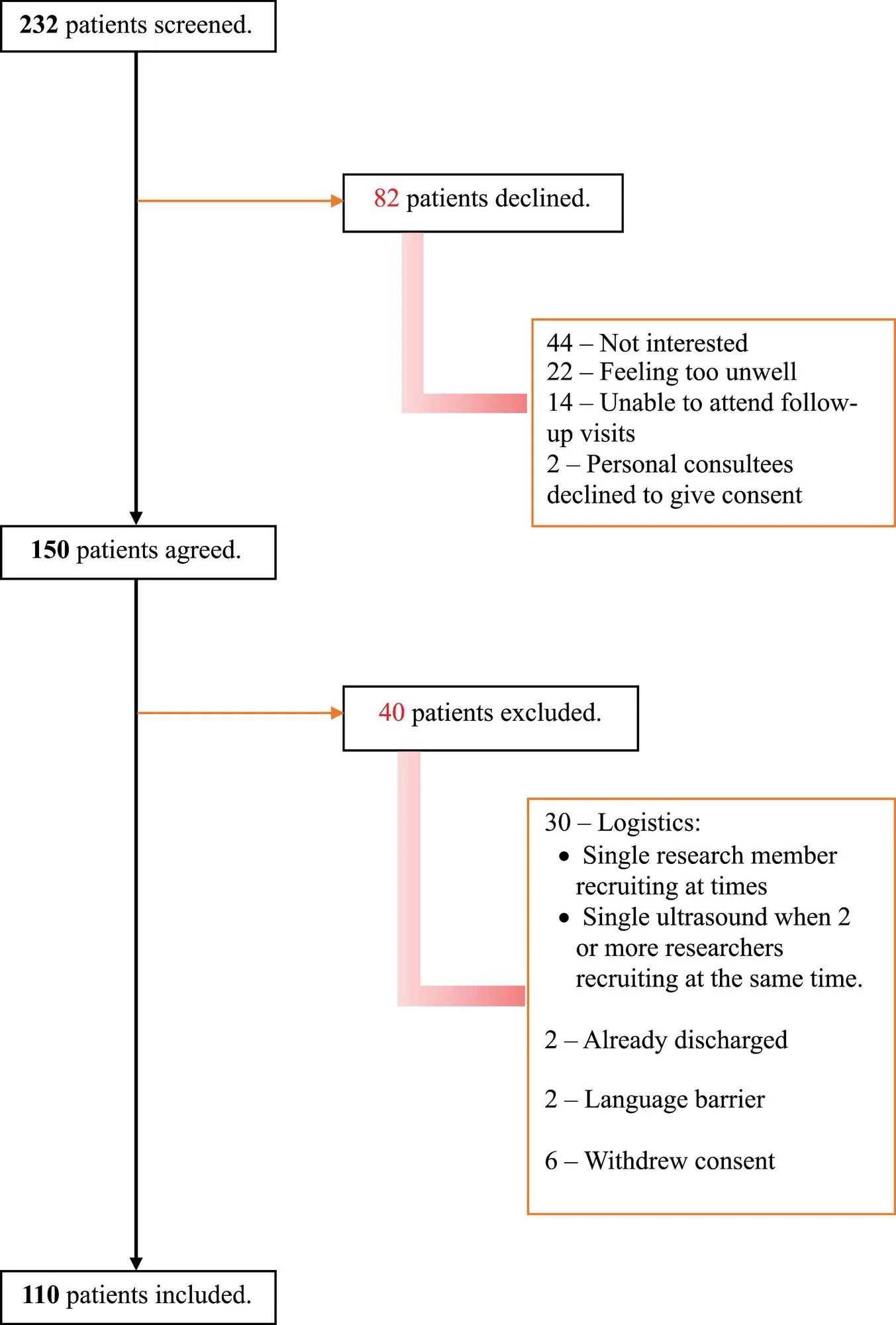
Flowchart of recruitment process into the TYSON study including reasons for exclusion.

A total of 232 participants were screened at admission, and 110 patients were included between October 2021 and December 2022. Figure 1 shows a flow diagram of the recruitment process into the TYSON study including reasons for exclusion.

**FIGURE 2:**
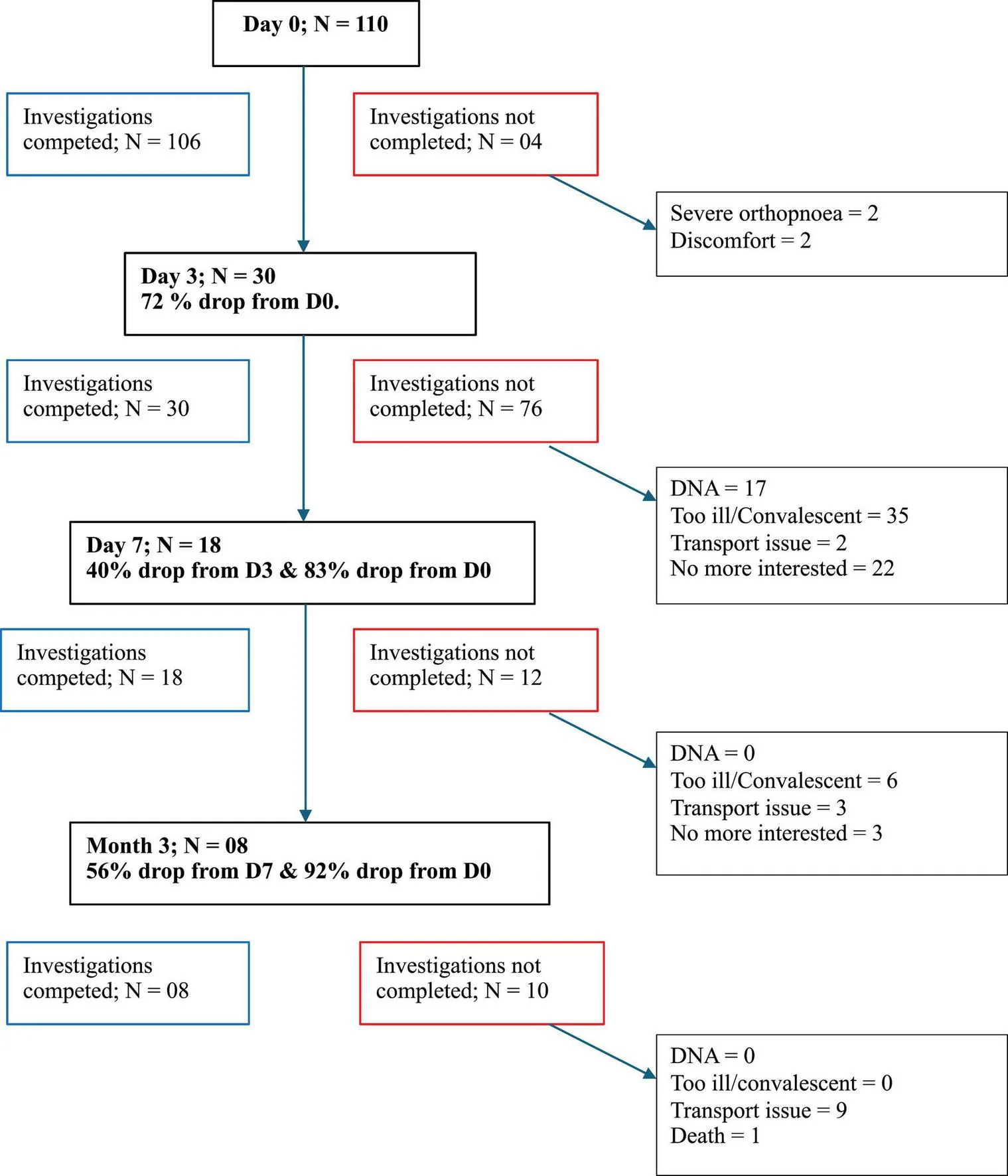
Participation drop out of our study. DNA, Did Not Attend; D0 = Day 0; D3 = Day 3; D7 = Day 7.

Data collection was complete in 96% (106) of participants at initial screening. Of those who did not complete all baseline data collection, four (3.6%) declined scanning of their thighs on admission (Day 0). Two participants reported severe breathlessness when lying down and the remaining two withdrew consent in the middle of the procedure because of discomfort. On Day three, only 30 participants remained in the study, with 76 (72%) withdrawing. On Day seven, 18 participants remained in the study, equating to an 83% withdrawal rate from Day 0. At 3 months, only eight participants agreed to data collection, equating to a 92% withdrawal rate from Day 0. Figure 2 summarizes the participation drop of our study population.

The two main reasons for non-participation in assessments were patients declining to re-attend hospital for follow-up visits due to post-acute illness fatigue, or discharge back to own home, nursing/care home with no transport availability to access the research clinic. The ultrasound equipment used was not portable and therefore home visits could not be offered with the equipment used for the study.

### Baseline assessment of sarcopenia and outcomes

At the baseline visit, all 106/106 (100%) patients had low muscle mass. 64/106 (60.4%) participants had associated low handgrip strength, therefore were diagnosed with sarcopenia. Of these, 60/64 (93.8%) confirmed sarcopenia had low physical performance and met the criteria for severe sarcopenia. 42/106 (39.6%) did not meet the diagnostic criteria for sarcopenia. None of the recruited patients were diagnosed with probable sarcopenia (low muscle strength alone).

Baseline characteristics are shown in Table 1. Patients with sarcopenia were mostly female (64% *vs.* 43%), older (78.1 years ± 7.1 *vs.* 74.7 years ± 6.4), and had a lower BMI (26.1 kgs/m^2^ ± 5.5 *vs.* 30 kgs/m^2^ ± 7.4), mid-arm circumference (26.8 cm ± 3.8 *vs.* 30.5 cm ± 4.6), calf circumference (33.6 cm ± 4.3 *vs.* 37.9 cm ± 6.6) and hemoglobin level (124.9 g/L ± 21.3 vs. 133 g/L ± 17), had a longer hospital stay and more of them died within 6-month compared to their non-sarcopenic counterparts.

**TABLE 1:** Demographics of participants included on admission (D0).

Characteristics	No sarcopenia, *N* = 42^1^	Sarcopenia, *N* = 64^1^
Age (years)	74.7 (6.4)	78.1 (7.1)
Sex		
Female	18 (43%)	41 (64%)
Male	24 (57%)	23 (36%)
Ethnicity		
White	42 (100%)	61 (95%)
Black	0	2 (3.1%)
Asian	0	1 (1.6%)
Body Mass Index (kgs/m^2^)	30.0 (7.4)	26.1 (5.5)
Unknown	0	2
Mid-arm circumference (cm)	30.5 (4.6)	26.8 (3.8)
Unknown	2	2
Calf circumference (cm)	37.9 (6.6)	33.6 (4.3)
Unknown	2	2
Nutritional status		
Normal nutritional status	8 (19%)	3 (5.2%)
At-risk of malnutrition	23 (55%)	32 (55%)
Malnourished	11 (26%)	23 (40%)
Unknown	0	6
Falls in last 12 months		
Less than 3	34 (81%)	44 (69.0%)
3 and more	8 (19%)	20 (31%)
Smoking status		
Non-smoker	12 (29%)	12 (19%)
Current smoker	10 (24%)	9 (14%)
Ex-smoker	16 (38%)	31 (48%)
Unknown	4	12
CRP	33.5 (50.1)	49.4 (53.0)
Unknown	0	4
Hemoglobin (dg/L)	133.0 (17.0)	124.9 (21.3)
Unknown	0	2
Early warning score	2.3 (2.6)	3.0 (2.8)
Muscle mass (mm)	5.1 (1.3)	4.2 (1.5)
Handgrip strength (kgs)	31.8 (4.7)	17.4 (6.2)
Gait speed (m/s)	0.4 (0.4)	0.3 (0.2)
Stroke		
Yes	5 (12%)	8 (13%)
No	33 (79%)	43 (67%)
Unknown	4	13
Myocardial infarction		
Yes	11 (26%)	8 (13%)
No	27 (64%)	43 (67%)
Unknown	4	13
Congestive cardiac failure		
Yes	7 (17%)	8 (13%)
No	31 (74%)	43 (67%)
Unknown	4	13
Diabetes mellitus		
Yes	13 (31%)	12 (19%)
No	25 (60%)	39 (61%)
Unknown	4	13
COPD		
Yes	12 (29%)	17 (27%)
No	26 (62%)	34 (53%)
Unknown	4	13
Cancer (all origin)		
Yes	4 (9.5%)	10 (16%)
No	34 (81%)	41 (64%)
Unknown	4	13
Length of stay (days)	7.0 (7.1)	10.6 (15.6)
In-patient death		
Yes	2 (4.8%)	4 (6.3%)
No	40 (95%)	60 (94%)
7-day readmission		
Yes	39 (93%)	61 (95%)
No	3 (7.1%)	3 (4.7%)
30-day readmission		
Yes	11 (26%)	14 (22%)
No	31 (74%)	50 (78%)
Death at 3-month		
Yes	3 (7.1%)	12 (19%)
No	39 (93%)	52 (81%)
Death at 6-month		
Yes	3 (7.1%)	23 (36%)
No	39 (93%)	41 (64%)

In a multiple logistic regression model adjusted for age, nutritional assessment, and hemoglobin level on admission, the presence of sarcopenia at hospital admission was associated with a five-fold increase in the odds of death at 6-month (OR 5.94, 95% CI 1.66–28.6) as shown in Table 2. Multivariable analyses for in-patient and 3-month mortality were limited by small event numbers. They should be interpreted as exploratory and used to inform the design of a future definitive trial.

**TABLE 2:** Exploratory impact of sarcopenia on mortality, adjusted for age, nutritional assessment, and hemoglobin level on admission.

Characteristic	In-patient mortality		3-month mortality		6-month mortality	
	OR^1^	95%CI^1^	OR^1^	95%CI^1^	OR^1^	95%CI^1^
Sarcopenia status						
No sarcopenia	–	–	–	–	–	–
Sarcopenia	0.53	0.06, 4.99	2.26	0.56, 11.4	5.94	1.66, 28.6
Age (years)	1.05	0.92, 1.23	1.08	0.98, 1.20	1.05	0.96, 1.15
Mini-nutritional assessment	0.75	0.53, 1.00	0.82	0.65, 1.00	0.77	0.62, 0.92
Hemoglobin level	0.99	0.94, 1.04	1.03	1.00, 1.07	1.02	0.99, 1.05

^1^OR = odds ratio; CI = confidence interval.

Only age was a predictor for 30-day readmission (Supplementary Table 1).

For every unit increase in the MNA score, there was a lesser risk of sarcopenic patients dying within 6-month (OR 0.77, 95% CI 0.62–0.92).

**FIGURE 3:**
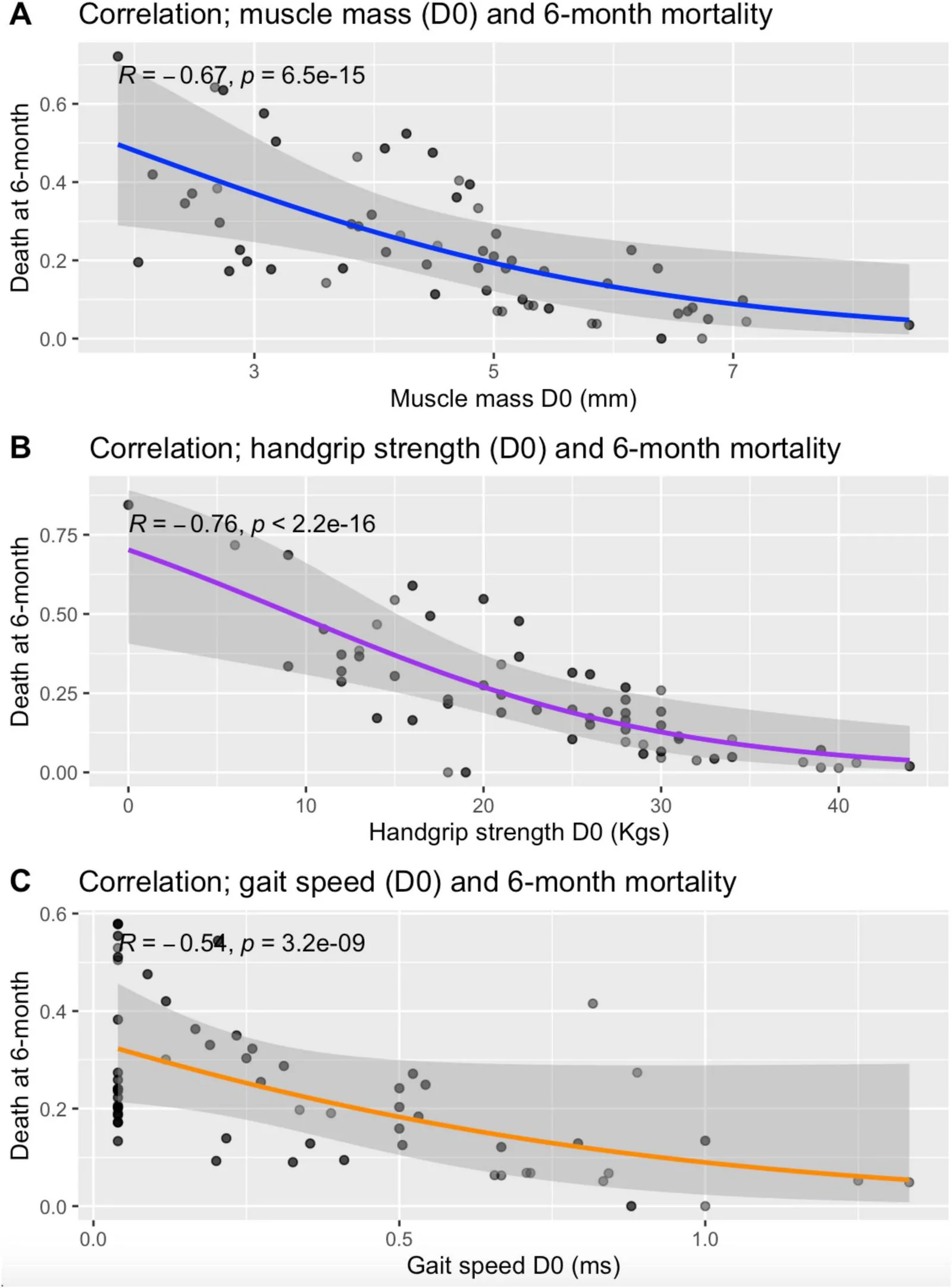
Predicted probability of 6-month mortality from logistic regression models including muscle mass **(A)**, muscle strength **(B)**, and gait speed **(C)** measured at admission (*n* = 106).

Figure 3 illustrate the correlation between the measures of muscle function on admission and the mortality at 6-month. Supplementary Table 2 highlights the difference for sarcopenic and non-sarcopenic patients.

Higher muscle mass, higher muscle strength and faster gait speed were all associated with a lower probability of dying 6 months post admission.

### Changes in patient demographics across sarcopenia measurements

#### Day 3 and 7 assessments

A smaller cohort of patients had repeated measures at Day 3 and Day 7.

The demographics of patients who presented at visits time-points D0, D3 and D7 are shown in Supplementary Table 2a.

There were no differences observed between those with and without a D3 visit in terms of age, sex, or ethnicity. Participants who completed the D3 visit had higher BMI (29.7 kgs/m^2^ ± 6.9 vs. 26.8 kgs/m^2^± 6.3), higher muscle mass (5.2 mm ± 1.4 *vs*. 4.3 mm ± 1.5) and greater handgrip strength on admission (26.3 kgs ± 10.5 *vs*. 21.9 kgs ± 8.2). Gait speed did not differ between the groups. The results are highlighted in Supplementary Table 2b.

The 18 (17%) participants who attended at Day 7 were younger (71.0 years ± 4.8 *vs.* 78.0 years ± 6.8), had a better nutritional status (28% *vs.* 7.3%) and were more likely to be female (83% *vs.* 50%). Most of them presented with less than 2 falls in the year preceding their admission. No difference was observed in ethnicity or muscle mass. We also noted they had higher handgrip strength (28.1 kgs ± 9.6 *vs.* 22.1 kgs ± 8.7) and faster gait speed (0.6 m/s ± 0.4 *vs.* 0.3 m/s ± 0.3) on admission as detailed in Supplementary Table 2c.

Our findings suggest that patients with better baseline muscle characteristics and those who were younger, stronger, and more mobile were more likely to agree to participate in the follow up visits, creating a significant attrition bias.

### Dynamic changes of sarcopenia

Characteristics of the stable participants who completed repeated assessments are summarized in Supplementary Table 3. These participants tended to be younger, have higher baseline muscle strength and better nutritional status than those who withdrew earlier in the study, consistent with a notable attrition bias mentioned earlier. As such, this study does not reliably assess changes in sarcopenia over time, these patterns should be interpreted as illustrative only and cannot be generalized to the wider cohort.

Participants were labeled as “stable” if there were no changes to sarcopenia status or severity; “improved,” if the sarcopenia status improved across visits and “deteriorated” if the sarcopenia status worsened across visits. Most patients were stable and showed no change in their sarcopenia status.

Four patients deteriorated from D3 to D7. They were all ex-smoker, Caucasian women with malnutrition (MNA < 17, ranging from 14.5 to 16) and mild anemia (Hb ranging from 111 to 118).

## Discussion

This study is one of the first to prospectively evaluate the prevalence of sarcopenia amongst older patients admitted in acute medical care unit and the feasibility of measuring dynamic changes during the acute admission and after discharge.

While almost 50% of people screened agreed to take part, which compares to other reported feasibility studies (32–34), and over 90% completed all baseline assessments, longitudinal assessments were harder to complete, with the majority of patients withdrawing at day 3, 7 assessments and very few returning for the 3-month assessment. Reasons registered were mainly the difficulties in returning to the hospital; the state of convalescence demotivating them to return for the previously agreed appointment; disinterest with study leading to their disengagement, or new admission into an institutionalized establishment. The retention and attrition bias toward more robust participants is clear. Possible ways of overcoming these issues and improving the participation rate of these older and vulnerable people in future studies include domicile visits, removing the burden of attending hospital, although different (portable) equipment would be needed to facilitate this. The study shows it is feasible to assess baseline sarcopenia, but longitudinal measurements for patients both who remain in hospital and especially those who are discharged home is challenging, in particular for those with more severe sarcopenia. Other studies have measured changes in sarcopenia but in different cohorts. Trevisan et al. reported 12-year sarcopenia trajectories measured every 3 years as part of a Swedish National Ageing cohort study, in 3219 community-dwelling patients aged ≥60 years old. This study demonstrated that while most patients progressed from no to having sarcopenia over the 12 years, some transitioning between having to not having sarcopenia was possible. The study also demonstrated poorer outcomes in patients with sarcopenia (35). Welch et al. studied a mixed cohort of 79 surgically (elective and emergency) and medically admitted participants. Similar to our acute medical cohort, medical patients in the Welch study had poorer physical function, a higher prevalence and severity of sarcopenia. Also similar to our study, Welch et al additionally reported higher drop-out rates in the medical cohort, with 87% retention in the elective surgical cohort compared to an approximately 50% retention rate in the medical cohort (36).

Sarcopenia was highly prevalent in acutely unwell adults aged ≥65 years on admission to hospital in the current study, with 60% presenting with a confirmed diagnosis and the majority being classified as having severe sarcopenia due to their associated low physical performance using the EWGSOP2 criteria. Most of the studies in the literature report a lesser rate, widely varying between 13% and 40%. This is mostly attributed to the difference in the sarcopenia classifications and cut-offs used between the reported studies and the population of patients included, with none looking at an acute medical unit population. Reported studies have included cohorts such as outpatients or community-dwelling patients (37, 38); patients aged <65 years (37, 39); patients with specific diseases such as chronic kidney disease (40–42), chronic obstructive pulmonary disease or COPD (43), stroke (44), diabetes (45) or patients with cancer (46). One meta-analysis conducted by Jiang et al. including 17 eligible studies and 3582 patients with mechanical ventilation, looking at the pooled prevalence of sarcopenia, found that 43% of their population was diagnosed with the condition and this had a negative impact on patients’ prognosis (47). Our study suggests acute medical admissions form a concentrated cohort of sarcopenic patients compared to other populations.

Our study showed that the presence of sarcopenia on admission was associated with a more than fivefold increase in the odds of mortality at 6-month. The results also highlight the possibility of a negative correlation between 6-month survival and muscle mass, muscle strength and gait speed measured on admission and an inverse relationship between nutritional status of our older patients and sarcopenia. Identifying sarcopenia in these patients is of potential significance, since it identifies a cohort at risk of significantly poorer outcomes and a cohort who may benefit most from interventions to address sarcopenia which may improve these outcomes. Many interventions involving a multi-disciplinary team have proven effective in preventing further muscle loss and functional decline. These include early physical mobilization, ranging from minimizing bed rest by sitting out as much as possible to undergoing a structured physiotherapy-led programme with tailored exercises to help strengthen the affected muscles; nutritional support with dieticians, including protein and vitamin D replacement when necessary.

Our study also looked at the dynamic changes of sarcopenia over time in older adults who had survived a recent unplanned admission. Although our longitudinal numbers were small and biased toward a less sarcopenic group, 89% (43/49) of our sub-population remained stable with 74.4% (32/43) of them non-sarcopenic and 25.6% (11/43) sarcopenic during the 7 days of their admission.

Diagnosing sarcopenia through an assessment of muscle function and muscle mass may not be feasible across all admitted patients, even when stratified by age. However, patients with sarcopenia on admission and those at risk of developing it can be identified with various screening tools (48) including the SARC-F questionnaire (49) which should be less burdensome to complete. Sarcopenia can also be identified during comprehensive geriatric assessment (CGA) delivered by qualified and trained professionals looking after older adults (50, 51).

This feasibility study has important strengths. It identified a group of patients with a high prevalence of sarcopenia and showed the importance of this measure as a predictor of poor outcomes. The study offers important learnings about the difficulties in gaining longitudinal data from an older and more sarcopenic and frail cohort without offering domicile visits and thus using portable equipment. This would be important in developing further studies of dynamic short-term trajectories in sarcopenia especially if interventions were planned. The study has important limitations, being based in one hospital setting (albeit it one that serves a diverse and large population) and the limited nature of the longitudinal data. Based on the former, an external validation of our results is needed.

## Conclusion and future directions

Studying sarcopenia in acutely admitted medical patients is possible, but serial follow up is challenging, with creative measures needed to facilitate participation.

Sarcopenia is associated with increased mortality in older people admitted to an acute medical unit. Muscle mass, muscle strength and gait speed at admission also correlate with 6-month mortality. Assessing sarcopenia in acute medical units may help to identify, early on during the admission, individuals at high risk of worse hospital outcomes. Further studies, ideally multicentre and with larger sample sizes are needed to (1) determine whether the difference in the clinical outcomes seen in our study are statistically significant, evaluate (2) sarcopenia trajectories and (3) the impact of any tailored interventions.

## Data Availability

The raw data supporting the conclusions of this article will be made available by the authors, without undue reservation.
