# Supplementary material for: Sarcopenia in older adults admitted to acute medical unit: a prospective observational study of feasibility and clinical outcomes
Source: Front Med (Lausanne). 2026 Aug 20;13:1862604. doi: 10.3389/fmed.2026.1862604 (PMC13539133; doi:10.3389/fmed.2026.1862604)
Supplement: Supplementary file 1 [file Table_1.docx]

Supplement 1: Exploratory impact of sarcopenia on hospital readmission, adjusted for age, sex, ethnicity, nutritional assessment, and haemoglobin level on admission.

| Characteristic | 7-day Readmission | | | 30-day Readmission | |
| --- | --- | --- | --- | --- | --- |
|  | **OR*^1^*** | **95%CI***^1^* | **OR***^1^* | | **95%CI***^1^* |
| Sarcopenia status |  |  |  | |  |
| *No sarcopenia* | – | – | – | | – |
| *Sarcopenia* | 0.28 | 0.03, 2.18 | 0.84 | | 0.29, 2.39 |
| Age (years) | 1.14 | 0.99, 1.38 | 1.09 | | 1.01, 1.19 |
| Sex |  |  |  | |  |
| *Female* | – | – | – | | – |
| *Male* | 3.04 | 0.34, 40.8 | 0.84 | | 0.29, 2.35 |
| Ethnicity |  |  |  | |  |
| *White* | – | – | – | | – |
| *Asian* | 0.00 |  | 0,00 | |  |
| *Black* | 0.00 |  | 0.00 | |  |
| Mini-Nutritional Assessment | 0.88 | 0.64, 1.16 | 1.04 | | 0.90, 1.20 |
| Haemoglobin level | 0.97 | 0.91, 1.03 | 1.02 | | 1.00, 1.05 |
| *^1^*OR = Odds Ratio; CI= Confidence interval. | | | | | |

Supplement 2a: Description of patients at subsequent visits (D3 and D7) compared to admission (D0)

| Characteristics | D0, N = 106*^1^* | D3, N = 30*^1^* | D7, N = 18*^1^* |
| --- | --- | --- | --- |
| Age (Years) | 76.8 (7.0) | 76.4 (7.5) | 71.0 (4.8) |
| Sex |  |  |  |
| *Female* | 59 (56%) | 18 (60%) | 15 (83%) |
| *Male* | 47 (44%) | 12 (40%) | 3 (17%) |
| Ethnicity |  |  |  |
| *White* | 103 (97%) | 29 (97%) | 17 (94%) |
| *Black* | 2 (1.9%) | 0 (0%) | 0 (0%) |
| *Asian* | 1 (0.9%) | 1 (3.3 %) | 1 (5.6%) |
| Body Mass Index (kg/m^2^) | 27.7 (6.6) | 29.7 (6.9) | 29.0 (7.5) |
| *Unknown* | *2* | *0* | *0* |
| Mid-Arm Circumference (cm) | 28.2 (4.5) | 29.4 (5.2) | 30.0 (5.6) |
| *Unknown* | *4* | *2* | *2* |
| Calf circumference (cm) | 35.3 (5.7) | 36.8 (8.1) | 36.6 (6.2) |
| *Unknown* | *4* | *2* | *2* |
| Nutritional status |  |  |  |
| *Normal nutritional status* | 11 (11%) | 5 (17%) | 5 (28%) |
| *At-risk of malnutrition* | 55 (55%) | 17 (57%) | 7 (39%) |
| *Malnourished* | 34 (34%) | 8 (27%) | 6 (33%) |
| *Unknown* | *6* | *0* | *0* |
| Falls in last 12 months |  |  |  |
| *Less than 3* | 78 (74%) | 26 (87%) | 16 (89%) |
| *3 and more* | 28 (26%) | 4 (13%) | 2(11%) |
| CRP | 42.9 (52.2) | 35.9 (54.0) | 22.6 (23.8) |
| *Unknown* | *4* | *0* | *0* |
| Haemoglobin (dg/L) | 128.2 (20.0) | 123.9 (20.1) | 127.5 (14.4) |
| *Unknown* | *2* | *0* | *0* |
| Early Warning Score | 2.7 (2.7) | 3.3 (2.8) | 1.4 (1.3) |
| Muscle mass (mm) | 4.6 (1.5) | 4.8 (1.3) | 4.9 (1.1) |
| Handgrip strength (kgs) | 23.1 (9.1) | 27.8 (11.2) | 28.6 (8.0) |
| Gait speed (m/s) | 0.3 (0.3) | 0.6 (0.5) | 0.7 (0.6) |
| Sarcopenia Diagnosis |  |  |  |
| *No Sarcopenia* | 42 (40%) | 19 (63%) | 13 (72%) |
| *Sarcopenia* | 64 (60%) | 11 (37%) | 5 (28%) |
| Length of Stay (days) | 9.2 (13.0) | 7.6 (5.6) | 16.3 (26.6) |
| In-patient death |  |  |  |
| *Yes* | 6 (6%) | 4 (13%) | 0 (0%) |
| *No* | 100 (94%) | 26 (87%) | 18 (100%) |
| 7-day Readmission |  |  |  |
| *Yes* | 6 (6%) | 4 (13%) | 0 (0%) |
| *No* | 100 (94%) | 26 (87%) | 18 (100%) |
| 30-day Readmission |  |  |  |
| *Yes* | 25 (24%) | 8 (27%) | 0 (0%) |
| *No* | 81 (76%) | 22 (73%) | 18 (100%) |
| Death at 3-Month |  |  |  |
| *Yes* | 15 (14%) | 6 (20%) | 0 (0%) |
| *No* | 91 (86%) | 24 (80%) | 18 (100%) |
| Death at 6-Month |  |  |  |
| *Yes* | 26 (25%) | 7 (23%) | 2 (11%) |
| *No* | 80 (75%) | 23 (77%) | 16 (89%) |
| *^1^*Mean (SD); n(%). | | | |

Supplement 2b: Baseline characteristics of patients presenting at D3visits compared to those who did not attend from admission.

| Characteristics | D3 visit, N = 30*^1^* | No D3 visit, N = 76*^1^* |
| --- | --- | --- |
| Age (Years) | 76.4 (7.5) | 76.9 (6.9) |
| Sex |  |  |
| *Female* | 18 (60%) | 41 (54%) |
| *Male* | 12 (40%) | 35 (46%) |
| Ethnicity |  |  |
| *White* | 29 (97%) | 74 (97%) |
| *Black* | 0 (0 %) | 2 (2.6%) |
| *Asian* | 1 (3.3%) | 0 (0 %) |
| Body Mass Index (kg/m^2^) | 29.7 (6.9) | 26.8 (6.3) |
| *Unknown* | *0* | *2* |
| Mid-Arm Circumference (cm) | 29.4 (5.2) | 27.8 (4.2) |
| *Unknown* | *2* | *2* |
| Calf circumference (cm) | 36.8 (8.1) | 34.7 (4.4) |
| *Unknown* | *2* | *2* |
| Nutritional status |  |  |
| *Normal nutritional status* | 5 (17%) | 6 (8.6%) |
| *At-risk of malnutrition* | 17 (57%) | 38 (54%) |
| *Malnourished* | 8 (27%) | 26 (37%) |
| *Unknown* | *0* | *6* |
| Haemoglobin (dg/L) | 123.9 (20.1) | 129.9 (19.8) |
| *Unknown* | *0* | *2* |
| Muscle mass (mm) | 5.2 (1.4) | 4.3 (1.5) |
| Handgrip strength (kgs) | 26.3 (10.5) | 21.9 (8.2) |
| Gait speed (m/s) | 0.4 (0.4) | 0.3 (0.3) |

Supplement 2c: Baseline characteristics of patients presenting at D7 visit compared to those who did not attend from admission.

| Characteristics | D7 visit, N = 18*^1^* | No D7 visit, N = 88*^1^* |
| --- | --- | --- |
| Age (Years) | 71.0 (4.8) | 78.0 (6.8) |
| Sex |  |  |
| *Female* | 15 (83%) | 44 (50%) |
| *Male* | 3 (17%) | 44 (50%) |
| Ethnicity |  |  |
| *White* | 17 (94%) | 86 (98%) |
| *Black* | 0 (0 %) | 2 (2.3%) |
| *Asian* | 1 (5.6%) | 0 (0 %) |
| Body Mass Index (kg/m^2^) | 29.0 (7.5) | 27.4 (6.4) |
| *Unknown* | *0* | *2* |
| Mid-Arm Circumference (cm) | 30.0 (5.6) | 27.9 (4.2) |
| *Unknown* | *2* | *2* |
| Calf circumference (cm) | 36.6 (6.2) | 35.0 (5.6) |
| *Unknown* | *2* | *2* |
| Nutritional status |  |  |
| *Normal nutritional status* | 5 (28%) | 6 (7.3%) |
| *At-risk of malnutrition* | 7 (39%) | 48 (59%) |
| *Malnourished* | 6 (33%) | 28 (34%) |
| *Unknown* | *0* | *6* |
| Haemoglobin (dg/L) | 127.5 (14.4) | 128.3 (21.0) |
| *Unknown* | *0* | *2* |
| Muscle mass (mm) | 4.9 (1.1) | 4.5 (1.6) |
| Handgrip strength (kgs) | 28.1 (9.6) | 22.1 (8.7) |
| Gait speed (m/s) | 0.6 (0.4) | 0.3 (0.3) |

Supplement 3: Descriptive characteristics of the stable sub-group population.

| Characteristics | NS, D0 – D3 | S, D0 – D3 | NS, D3 – D7 |
| --- | --- | --- | --- |
|  | **N = 19** | **N = 10** | **N = 13** |
| Age (Years) | 72.4 (5.2) | 84.1 (5.2) | 69.5 (4.5) |
| Sex |  |  |  |
| *Female* | 10 (53%) | 8 (80%) | 10 (77%) |
| *Male* | 9 (47%) | 2 (20%) | 3 (23%) |
| Ethnicity |  |  |  |
| *White* | 19 (100%) | 9 (90%) | 13 (100%) |
| *Black* | 0 | 0 | 0 |
| *Asian* | 0 | 1 (10%) | 0 |
| Body Mass Index (kg/m^2^) | 31 (6.8) | 26.1 (4.9) | 30.6 (8.2) |
| Mid-Arm Circumference (cm) | 31.1 (4.4) | 26 (5) | 31.8 (5.8) |
| *Unknown* | *2* | – | *2* |
| Calf circumference (cm) | 39.9 (7.7) | 30.8 (5.1) | 38.7 (6.4) |
| *Unknown* | *2* |  | *2* |
| Nutritional status |  |  |  |
| *Normal nutritional status* | 4 (21%) | 1 (10%) | 4 (31%) |
| *At-risk of malnutrition* | 11 (58%) | 5 (50%) | 7 (54%) |
| *Malnourished* | 4 (21%) | 4 (40%) | 2 (15%) |
| Falls in last 12 months |  |  |  |
| *Less than 3* | 17 (89%) | 8 (80%) | 13 (100%) |
| *3 or more* | 2 (11%) | 2 (20%) | 0 |
| Smoking status |  |  |  |
| *Non-smoker* | 5 (26%) | 1 (10%) | 5 (38%) |
| *Current smoker* | 6 (32%) | 1 (10%) | 2 (15%) |
| *Ex-smoker* | 4 (21%) | 8 (80%) | 4 (31%) |
| *Unknown* | *4* | – | *2* |
| *Co-Morbidities* | *NA = 4* |  | *NA = 2* |
| Stroke |  |  |  |
| *Yes* | 4 (21%) | 2 (20%) | 2 (15%) |
| *No* | 11 (58%) | 8 (80%) | 9 (69%) |
| Myocardial Infarction |  |  |  |
| *Yes* | 6 (32%) | 4 (40%) | 2 (15%) |
| *No* | 9 (47%) | 6 (60%) | 9 (69%) |
| Congestive cardiac failure |  |  |  |
| *Yes* | 6 (32%) | 1 (10%) | 2 (15%) |
| *No* | 9 (47%) | 9 (90%) | 9 (69%) |
| Diabetes Mellitus |  |  |  |
| *Yes* | 0 | 3 (30%) | 11 (85%) |
| *No* | 15 (79%) | 7 (70%) | 0 |
| COPD |  |  |  |
| *Yes* | 5 (26%) | 2 (20%) | 3 (23%) |
| *No* | 10 (53%) | 8 (80%) | 8 (62%) |
| Cancer (all origin) |  |  |  |
| *Yes* | 2 (11%) | 2 (20%) | 2 (15%) |
| *No* | 13 (68%) | 8 (80%) | 9 (69%) |
| CRP | 44.5 (59.2) | 22.9 (43.7) | 16.6 (20.1) |
| Haemoglobin (dg/L) | 125.3 (18.9) | 119.6 (22.9) | 130.6 (15.4) |
| Muscle mass (mm) | 5.6 (1.2) | 4.6 (1.6) | 5.3 (0.7) |
| Handgrip strength (kgs) | 32.8 (4.5) | 15.1 (8.4) | 33.3 (4.3) |
| Gait speed (m/s) | 0.5 (0.4) | 0.2 (0.3) | 0.6 (0.4) |
